# The Impact of CRISPR-Cas9 on Age-related Disorders: From Pathology to Therapy

**DOI:** 10.14336/AD.2019.0927

**Published:** 2020-07-23

**Authors:** Allen Caobi, Rajib Kumar Dutta, Luis D Garbinski, Maria Esteban-Lopez, Yasemin Ceyhan, Mickensone Andre, Marko Manevski, Chet Raj Ojha, Jessica Lapierre, Sneham Tiwari, Tiyash Parira, Nazira El-Hage

**Affiliations:** ^1^Departments of Immunology and Nano-medicine,; ^2^Human and Molecular Genetics and; ^3^Cell Biology and Pharmacology, Herbert Wertheim College of Medicine, Florida International University, Miami, Florida, USA.

**Keywords:** gene-editing, aging, CRISPR-Cas9, neurodegeneration, cancer, alternative medicine

## Abstract

With advances in medical technology, the number of people over the age of 60 is on the rise, and thus, increasing the prevalence of age-related pathologies within the aging population. Neurodegenerative disorders, cancers, metabolic and inflammatory diseases are some of the most prevalent age-related pathologies affecting the growing population. It is imperative that a new treatment to combat these pathologies be developed. Although, still in its infancy, the CRISPR-Cas9 system has become a potent gene-editing tool capable of correcting gene-mediated age-related pathology, and therefore ameliorating or eliminating disease symptoms. Deleting target genes using the CRISPR-Cas9 system or correcting for gene mutations may ameliorate many different neurodegenerative disorders detected in the aging population. Cancer cells targeted by the CRISPR-Cas9 system may result in an increased sensitivity to chemotherapeutics, lower proliferation, and higher cancer cell death. Finally, reducing gene targeting inflammatory molecules production through microRNA knockout holds promise as a therapeutic strategy for both arthritis and inflammation. Here we present a review based on how the expanding world of genome editing can be applied to disorders and diseases affecting the aging population.

## CRISPR-CAS9: Introduction, Mechanism and Importance

**Figure 1.**
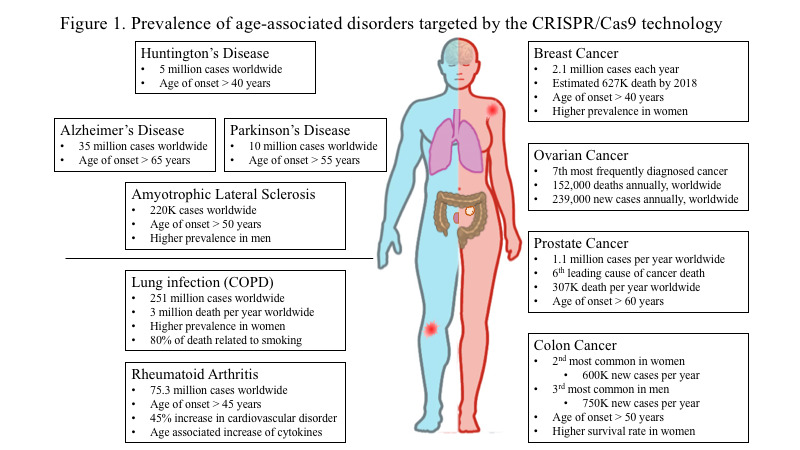
**Prevalence of age-associated disorders that can be targeted by the CRISPR-Cas9 technology**. Schematic diagram of the health burden associated with increased life expectancy in men (depicted in blue color) and women (depicted in pink color). Neurodegenerative disorders, cancer, metabolic and inflammatory diseases are among the most prevalent age-related pathologies affecting the growing population.

Throughout the world, people over 60 years of age are becoming an increasingly large percentage of the total population. In the year 2012, the estimated population over the age of 60 was about 43.1 million or less than 20% of the U.S. population. However, the projected number of the American population reaching the age of 60 and older by the year 2050, is expected to reach around 83 million or about 25 - 30% of the U.S. population. This information is according to the U.S. Census Bureau [1-3]. With the increase in human life expectancy comes an increase in the prevalence of age-related pathologies and health burdens in the aging population (Fig. 1) [1]. Neurodegenerative disorders, cancer, metabolic and inflammatory diseases are some of the most prevalent age-related pathologies affecting this growing population [1, 2, 4-7]. In this new era of targeting therapeutics, gene editing promising tool against a plethora of diseasesIn tiis a promising tool against a plethora of diseases [8, 9]. The most imperative and critical requirement to understand the pathological mechanisms of the diseases is understanding the functions of a gene or multiple genes in primary human cells and targeting them.

Since it was first discovered in *Caenorhabditis elegans*, RNA interference (RNAi) was used as a mechanism to knockdown genes of interest [10]. However, this technology presents multiple drawbacks, including incomplete or insufficient knockout and off-target effects [11]. The clustered regularly interspaced palindromic repeats- (CRISPR-) associated (Cas) protein 9 (known as CRISPR-Cas9) system targets and induces site-specific DNA double-strand breaks (DSBs) directed by a single-guide RNA (sgRNA) that enables the editing of the genome by adding, removing, or altering sections of the DNA sequences in a variety of species [12-14]. The concept of ene-editing by a complete knockout of a gene in human cells with minimal off-targeting events represents a powerful approach to study gene function and to discern the molecular mechanisms underlying complex human diseases with the ultimate goal of improving the quality of gene therapy studies [15]. This method has been employed to identify or investigate: cancer-associated gene function, revealing the role of numerous variants and the non-coding region in tumor development, epigenetic mechanisms, cancer risk via a genetic screen, the development of animal models, and as a potential cancer therapeutic tool [16]. There are three types of CRISPR-Cas9 gene editing systems [17]. The most studied system is the Type II, that was adapted from a naturally occurring genome editing system in the bacteria *Streptococcus pyogenes* [17]. Here we will briefly discuss the different forms of the CRISPR-Cas9 technology. However, an in-depth review of CRISPR-Cas9 cell entry and mechanism of action is beyond the scope of this review and already covered in great detail elsewhere [9, 18-27].

Unlike the bacterial adaptive immunity, the CRISPR-Cas9 gene-editing tool is less complex, as it requires only three components including the crRNA, trRNA, and Cas9 [13, 14, 17, 18, 28, 29]. CRISPR-Cas9 can be used to generate an RNA-programmable method of gene editing in eukaryotic cells, allowing gene knockouts via non-homologous end joining (NHEJ), or knock-ins via homology-directed repair (HDR) [17, 18]. By engineering a synthetic single-guide RNA (sgRNA), combined with crRNA and trRNA, a simpler two-component system is formed. Currently there are three variants of the Cas9 nuclease used in research [17, 18]. To generate indels, a wild-type Cas9 is used to generate DSBs which are repaired via either the NHEJ or the HDR pathway [17, 18]. Without a DNA template guiding repair, the error-prone NHEJ repair pathway introduces indels as a consequence of misaligned repair due to micro-homology, causing gene knockouts and frameshift mutations [17, 18]. Addition of a homologous DNA template to guide repair, allows usage of the HDR pathway, which significantly reduces indel mutations, while providing a gene-editing system with greater target specificity capable of inserting gene corrections by assisted recombination [17, 18]. Alternatively, if precise insertion/replacement of the DNA is desired, the addition of a donor template, homologous to the target locus, along with a mutant-form Cas9D10A will induce repair via the HDR pathway, as the enzyme will only possess nickase activity, resulting in the cleavage of a single DNA strand [30]. Deactivated Cas9 (dCas9), as a result of nuclease domain inactivation, can still function as a DNA-binding scaffold to either silence (CRISPRi) or activate (CRISPRa) gene expression [31, 32]. The two-component system may be packaged within either viral or non-viral vectors [19]. Retroviral, lentiviral, and adeno-associated viral vectors have been employed as CRISPR-Cas9 transporters to target cells [19]. Additionally, liposomes, nanoparticles, and cell-penetrating peptides (CPP) have also been used as a non-viral method for the delivery of CRISPR-Cas9 to target cells [19].

The DNA endonuclease Cas9 functions by binding to a sgRNA [13, 33]. The RNA sequence binds and directs Cas9 to the cleavage site; hence, rather then engineering a specific, single-use endonuclease, synthesis of the sgRNA combined with the expression of Cas9 can be used to create similarly specific double-strand breaks [13, 17, 33, 34]. This has allowed for the exploration of genome-wide loss and gain-of-function screens [35, 36], suppression and re-activation of signaling pathways and key effectors [37, 38], and identification of distinct genetic signatures [39], all of which will be discussed in the context of colorectal cancer, a disease strongly associated with an ever-increasing aging population [40].

**Table 1:** Therapeutic applications of CRISPR-Cas9 system in age-related disorders.

Disorders	Target Sites	Model	Advantages	Disadvantages	Refs
**Amyotrophic Lateral Sclerosis (ALS)**	*SOD1* and *FUS*	Human ALS patient fibroblast	Corrects the mutation A272C in SOD1 and G1566A in FUS.	Not clear if treatment after disease onset would be effective. Reduces the expression of both wild type and mutant gene.	(51)
	*tardbp* and *fus*	Zebrafish	Correction of missense mutations in these ALS-associated genes.	N/A^*^	(55)
**Alzheimer’s Disease (AD)**	Mutation in Amyloid Precursor Protein (*APP*)	Human	Disrupts expression of mutant APP.	Shortening the gRNA could lead to decreased on-target efficacy	(66; 75)
	*PSEN2*	Basal forebrain cholinergic neurons	Correction of the N141I mutation resulted in normalization of observed Aβ42/40 increase.	N/A^*^	(73)
	*PSEN2*	Human	Abolishes the electrophysiological deficit and restores the number of spikes and spike height.	N/A^*^	(73)
	*PSEN1*	Human	(c.236 T > C) mutation correction.	N/A^*^	(71)
	*PSEN1*	Human	(c.449C > T) mutation correction of the PSEN1 gene.	N/A^*^	(72)
	*APOE4*	Human	Converts APOE4 to APOE2 or E3. Effective in neutralizing the risk of AD.	N/A^*^	(68)
**Parkinson’s Disease (PD)**	*LRRK2*	Human	Corrects the p.G2019S mutation in LRRK2 and neurite complexity. Retained pluripotency of hiPSCs after gene editing.	N/A^*^	(102)
	*SNCA*	Human cell line	Corrects mutation in SNCA gene.	N/A^*^	(103)
**Colorectal Cancer (CRC)**	*PAR3L*	Human CaCO-2 Cells	KO results in reduced proliferation and induction of apoptosis of CRC cell line.	Study was limited to CRC cell lines, no primary cells used.	(38)
	*TP53*	Human colon adenocarcinoma-derived cell lines	Correction of mutations of TP53 at exon 3 and exon 10 may alter the malignant potential of these cells.	Not tested on all of the genomic mutations and clinical varieties of TP53.	(131)
	*APC*	Human and mouse organoids	Colonoscopy-guided mucosal injection used to deliver CRISPR-engineered organoids. Facilitates studying adenoma-carcinoma-metastasis progression.	Colonoscopy and specific surgical equipment are required.	(139)
	*KRAS*	Human cell lines	sgRNA library targeting protein-coding genes in *KRAS*-mutant CRC cell lines used to identify genes associated with reduced tumor growth.	N/A^*^	(36)
**Prostate Cancer**	*PD-1*	Phase I clinical trial	PD-1 knockdown of T cells in castration-resistant prostate cancer.	Confirmation of successful knockdown and a significant change in disease phenotype cannot yet be made, as the clinical trial is ongoing.	(152)
	*GPRC6A*	Human cell line	Reduces primary tumor growth.	N/A^*^	(148)
	Androgen receptor (*AR*) gene	Human Cell line	Restrains growth of androgen-dependent prostate cancer and potential therapeutic strategy for prostate cancer treatment.	Limited to androgen-dependent prostate cancer not androgen-independent prostate cancer.	(146)
	Transcription factor NANOG andpseudogene *NANOGP8*	Human cell line	Attenuates malignant potential and migration capability.	Knockout of both *NANOG1* and *NANOGP8* genes is lethal.	(150)
**Breast Cancer**	*HER2*	Human cell line	Inhibits cell growth and tumorgenicity.	Effects downstream MAPK/ERK and PI3K/AKT signaling cascades, in non-cancer cells.	(163)
	*Pten*	Mouse model	*Pten* silencing by lentiviral delivery results in development of invasive lobular breast cancer.	Lentiviral delivery causes immune response.	(161)
	*CDK8/19*	Human cell line	Suppress estrogen-induced gene expression in breast cancer.	N/A^*^	(169)
	*Ubr5*	Mice	Impairs tumor growth and metastasis.	N/A^*^	(170)
	*MIEN1*	Human epithelial breast cancer	Deletions of MIEN1 gene lead to the abrogation of breast cancer.	N/A^*^	(171)
**Ovarian Cancer**	*DNMT1*	Human ovarian cancer cell line (SKOV-3) and mice	Inhibition of tumor growth	N/A^*^	(175; 176)
	*MTH1*	A subcutaneous xenograft tumor model of SKOV3 cells in BALB/c nude mice	Apoptosis and genetic damage of cancerous cells resulting in tumor growth inhibition.	N/A^*^	(177)
	*miR-21*	Human ovarian cancer cell lines (SKOV-3 & OVCAR3)	Inhibition of the epithelial-to-mesenchymal transition (EMT) in ovarian cancer cells.	N/A^*^	(178)
	*PARP-1*	SKOV-3 cell line and a SKOV-3 xenograft BALB/C mice model	Increased cancer cell death	N/A^*^	(179)
Rheumatoid arthritis	*FOXP3*-associated genes	Human Regulatory T-cell (Treg)	Augmentation of the suppressive ability of Tregs via increased Treg stability. Insertion of chimeric antigen receptor (CAR) gene increased potency in cancer therapy.	N/A^*^	(181)
Lung infection	*MUC18*	Human primary airway epithelial cells (AECs)	Reduced IL-8 (proinflammatory chemokine) responses.	Mixed population of edited cells and phenotypic changes unrelated to the gene knockout.	(12)

Table 1 includes a list of diseases related to the aging population that the CRISPR technology has been used for. Included in the list are the target sites, models, advantages and obstacles of using CRISPR-Cas9 technology.

The fast-moving field of genome engineering has enabled the systematic interrogation of gene functions and the editing of DNA, modulating their function and potentially yielding gene therapies to treat genetic disease [9, 41]. The emerging genome editing technologies include transcription activator-like effector nucleases (TALENs), zinc-finger nucleases (ZFNs), and the RNA-guided CRISPR-Cas9 system [41]. Unlike its predecessors, the CRISPR-Cas9 system is highly specific, is effortless to design, and is compatible with multiplex and high-throughput gene editing of a multitude of cells and organisms [41]. These traits facilitate the use of this system for the treatment of genetic disease, with trials on mice and human cells successfully eradicating genetic diseases in not only mice, but also its progeny [42, 43]. In this review, we discuss how the expanding world of genome editing can be applied to disorders and diseases affecting the aging population. Specifically, focusing on benefits and disadvantages of using CRISPR-Cas9 technology in neurodegeneration, cancers and inflammatory diseases (Table 1).

## Amyotrophic lateral sclerosis (ALS) and gene therapy using the CRISPR-Cas9 system

**Figure 2.**
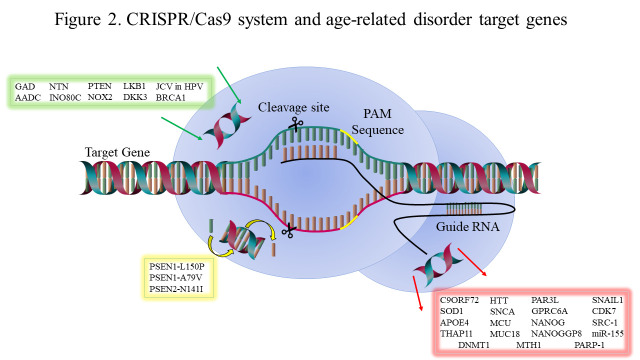
**CRISPR-Cas9 system and age-related disorder target genes**. Schematic representation of the CRISPR-Cas9 mediated genome editing and potential target genes associated with ALS, AD, PD, HD, cancers and inflammatory disorders. The functional gene may be inserted (green box), the mutated gene may be replaced with a wild-type gene (yellow box) or be removed altogether (red box).

The precedent for the development of CRISPR-Cas9 as a therapeutic tool against genetic disease has been set. However, the question remains whether this system can be employed to treat genetic disease associated with aging. Aging is described as a multifactor phenomenon, characterized by reduced cellular process and physiological functions, susceptible to several critical diseases and increased probability of death [1, 2, 44, 45]. Age-related disorders can range from a plethora of disorders including cancers to neurodegenerative disorders. Amyotrophic lateral sclerosis (ALS) and frontotemporal dementia (FTD) are caused by a hexanucleotide-repeat expansions in the *C9ORF72* gene [46]. In ALS, C9ORF72 is age-dependent and inherited in an autosomal dominant manner. ALS is a terminal neurodegenerative disease characterized by a progressive loss of motor neurons in the spinal cord and in the brain resulting in generalized weakness, paralysis, and eventual death from respiratory failure; additionally, ALS has pathobiological features in common with FTD [47, 48]. FTD is a progressive neurodegenerative disorder which typically presents with changes in social conduct, behavior, and personality [49]. Atrophy of the prefrontal and anterotemporal cortex has been linked to FTD [50]. Two other clinical manifestations of FTD, semantic dementia (SD) and progressive non-fluent aphasia (PNFA), primarily exhibit language dysfunction [49, 50]. The fact that both diseases are caused by expansions within a single gene implies that these diseases are phenotypic extremes of a single spectrum disorder [48]. The hexanucleotide-repeat expansions in the *C9ORF72* gene translates into aggregation-prone dipeptide-repeat (DPR) proteins, contributing to neurodegeneration [46]. Kramer et al, employed the CRISPR-Cas9 system to reduce expression of C9ORF72 DPR toxicity in human cells via gene-knockout screens against its enhancers and suppressors. This process elucidated candidate genes involved in chromatin modification, nucleocytoplasmic transport and RNA processing [46]. This demonstrated the potential of the CRISPR-Cas9 system in the identification of new candidate target genes, discovery of pathway roles with the ALS phenotype, and its capacity to be used as a therapeutic tool (Fig. 2).

Since the primary goal of using the CRISPR-Cas9 gene-editing tool is to treat disease, studies focusing on therapeutic aspect is on the rise. In a study by Wang et al, they have generated immune-pluripotent stem cells (iPSCs) of ALS patients carrying *SOD1* ^+/^*^A272C^* and *FUS* ^+/^*^G1566^* heterozygous mutations, implicated as a cause for familial ALS (FALS), and then corrected the aforementioned genes using the *CRISPR-*Cas9 system [51]. Given that SOD1 gene mutations account for about 20% of all inherited cases of ALS and previous studies demonstrating disease amelioration via RNAi-induced silencing of SOD1. In a study by Kennedy et al, they showed that silencing SOD1 resulted in an attenuation in disease symptoms that correlated with increased survival time in SOD1-transgenic mice [52]. One caveat of using the CRISPR-Cas9 system, regarding ALS therapeutics, lies in the potential off-target effects which must be addressed before clinical trials commence [51, 52]. Despite, this short-coming, the use of CRISPR-Cas9 as a gene therapy platform should result in a smooth transition from an exploratory identification of candidate genes in animal studies to eventual clinical trials [52, 53].

Currently, the use of animal models to validate the efficacy and safety of CRISPR-Cas9 as a genetic tool has significantly improved our understanding of disease pathophysiology with significant support in the development of preventative and therapeutic strategies [53]. Novel models developed using the CRISPR-Cas9 system have resulted in a renaissance across fields, facilitating studies such as those modelling neurodegenerative disease through mutations in endogenous genes by significantly reducing the amount of time and effort of generating mutant mice strains [53, 54].

To better understand ALS, the generation and use of animal model using the CRISPR-Cas9 system would expedite the development of potential therapeutic solution, since the condition is caused by an amalgamation of genetic and environmental factors [53]. The remaining cases of ALS are associated with mutations in various genes, such as in the fused/translocated liposarcoma (FUS) or the 43-kDa TAR DNA-binding protein (TARDBP) gene [53]. Several studies have already attempted to generate novel CRISPR-generated transgenic ALS animal models [54]. In fact, in the C9ORF72-deficient mice model, CRISPR-Cas9 was used to develop murine pathogenic variants of ALS to mimic multiple-mutation in genes coding different domains of a single protein in human [53]. In the ALS zebrafish model developed by Armstrong et al, they used the CRISPR-Cas9 system to introduce point mutations in the TARDBP and FUS genes; providing evidence that the generation of human disease induced by point mutations can be imitated with knock-in lines developed by homology-directed repair (HDR) following CRISPR-Cas9 [53, 55]. Remarkably, the CRISPR-Cas9 system was able to edit the endogenous gene, despite being expressed in a phylogenetically distant species [53, 55], which saved time and effort as genomic integration in zebrafish can be evaluated within two days [53, 55]. In another ALS-mouse model, CRISPR-Cas9 system was used to knocked-down the gene encoding insulin-like growth factor 1 (IGF1) [53, 56]. This allowed Lin et al, to observe the processes that are influenced by neuronal growth factor, in the context of ALS [53, 56]. Using novel animal models brings us closer to a potential therapeutic solution, as traditional therapies are symptom-based and therefore not able to completely eliminate the pathology underlying the clinical manifestations [53, 57]. Given that gene therapy permanently alters intracellular processes garners hope towards the eventual treatment of neurodegenerative diseases, which are currently incurable [53].

## Alzheimer’s disease (AD) and gene therapy using the CRISPR-Cas9 system

Alzheimer’s disease (AD) is a major neurodegenerative disease characterized by the formation of amyloid plaques and neurofibrillary tangles of hyperphosphorylated tau in the brain [58]. These aggregations of aberrant proteins cause progressive neuronal loss, severe cognitive impairment, and dementia that ultimately lead to the patient’s premature death [58, 59]. Despite efforts of the scientific and the healthcare communities to treat this devastating disease, there is still no cure for AD [60]. Today there are over 35 million people worldwide living with dementia and AD-like disorders and this number is projected to reach 114 million by 2050 [60, 61]. In the US alone, there is an estimated 5 million patients affected by the disease [62]. These statistics alone show the profound impact that AD has on our society and the urgent need to invest in research to improve therapeutic strategies. Genetic predisposition and lifestyle choices may contribute to AD development but age-related changes that leads to proteostasis and mitochondrial impairment in neuronal or supporting cells may be of greater impact in the development of the disease [63, 64]. AD is usually classified into two categories: early- and late-onset AD [65]. Familial or Early-onset AD covers a small subset of all AD cases, affecting patients as early as 30 years old [65]. Three genes, presenilin 1 (*PSEN1),* presenilin 2 *(PSEN2)*, and amyloid precursor protein (*APP*), have known pathogenic mutations that are strongly associated with the development of early-onset AD (Fig. 2); although mutations in additional genes may be involved in the pathology of the disease as well [65, 66]. Late-onset AD is the most common form of AD and it is considered sporadic, affecting the elderly (> 65 years old) [65]. The causes of the disease in this stage are not clear, but age-related changes appear to play a determinant role [65]. The gene *APOE4* is a potent risk factor for the development of late-onset AD through stimulating amyloid-β production and expressed in more than 60% of AD patients [65, 67, 68]. The current treatment for AD consists of cholinesterase inhibitors such as donepezil and the glutamate agonist memantine [69]. These drugs only ameliorate the symptoms, without stopping the course of the disease, underlining the critical need of finding new therapies for AD [69]*.*

CRISPR-Cas9 technology emerged as a feasible gene therapy for the treatment of AD. The recent development of a mitochondria-targeted CRISPR-Cas9 known as “mitoCas9” could be applied to edit mtDNA and remove or revert mutations that accumulate with age, impairing oxidative phosphorylation and increasing ROS production [70]. The mitoCas9 uses sgRNAs customized to target specific mutations on the mitochondrial genome leaving the nuclear DNA intact [70]. The CRISPR-Cas9 system using the sgRNAs was also successfully applied to correct A79V and L150P point mutations in the *PSEN1* gene of human-induced pluripotent stem cells (hiPSCs) derived from familial AD patients [71, 72]. These studies used Sanger sequencing and karyotyping to validate their CRISPR-Cas9-corrected mutations [71, 72]. Another example includes the correction of the *PSEN2* N141I mutation in an iPSC-derived neuronal model where the amyloid-β 42/40 ratio was normalized after gene editing treatment [73]. Ortiz-Virumbrales et al performed electrophysiological studies with various stimuli to determine functional restoration in the CRISPR-Cas9 corrected cells [73]. Future directions of this technology may include developing of CRISPR-Cas9 mediated gene editing to decrease the levels of APOE4 and amyloid-β in AD patients [74, 75]. Recently, an APP mutation has been identified as the cause for dominantly inherited AD in Swedish populations, resulting in very high amyloid-β levels due to increased β-secretase cleavage of the amyloid-β (Aβ) precursor protein [76]. Fibroblasts from human subjects carrying the mutation as well as transgenic mouse models were generated and used to prove the potential therapeutic application of CRISPR-Cas9 [76]. Co-injection of adeno-associated viral (AAV) vectors carrying coding sequences for the human mutant *APP^SW^* allele gRNA and Cas9 into the adult mouse hippocampus successfully disrupted the mutation in the *APP* gene [76]. Hippocampus and cerebellum isolated and sequenced post-mortem from mice determined that the CRISPR-Cas9 correction was achieved [76]. These examples demonstrate how CRISPR-Cas9 as an emerging technology can have wide application in the context of AD. One of the important factors to consider in gene therapy is the type of delivery vector, which must be specific for the target organ to avoid any undesired effects or systemic toxicity. In this regard, the use of recombinant AAV vectors is becoming relevant in the treatment of neurological disorders. The use of a chimeric AAV known as AAV2g9, showed a brain-specific CRISPR-Cas9-mediated gene deletion of the schizophrenia risk gene MIR137 in mice [77]. Overall, this is a promising result that could be translated to the gene therapy treatment of AD and other neurodegenerative diseases.

## Parkinson's disease (PD) and gene therapy using the CRISPR-Cas9 system

Aging is also a major risk factor for the development and advancement of idiopathic Parkinson’s disease (PD) [78]. In fact, after Alzheimer’s disease, Parkinson’s disease is considered the second most age-related, chronic, and aggressive neurodegenerative disease in the aging populations [78]. The pathology of Parkinson's disease (PD) is primarily characterized by deposition of Lewy bodies and Lewy neurites that contain α-synuclein, highly conserved protein throughout the central (CNS) and peripheral nervous systems (PNS) which leads to the irreversible loss of dopaminergic neurons in the substantia nigra pars compacta and eventual events attributed on the cerebral cortex [79, 80]. There are several theories regarding whether the pathological progression of PD is involved with aging, or whether aging increases the susceptibility to PD, or if there are other factors or processes involved with aging which exacerbate the progression of PD [81]. In addition to aging, several studies reported that exposure to environmental agents like pesticides, cigarette smoking, dietary factors, genetic predisposition, or alteration of mitochondrial function has also been found to significantly influence the occurrence and progression of Parkinson’s disease [78, 79, 82]. According to the National Institutes of Health (NIH), an estimated 500,000 people live with PD in the U.S. and it affects 1-2 individuals per 1000 people at any time, worldwide [83, 84]. Parkinson’s disease affects approximately 1% of individuals age 60 or older and no more than 3% of those older than 80 years old [85]. The overall age- and gender-adjusted incidence rate is (13.4:100,000), with a higher prevalence among males (19:100,000) than females (9.9:100,000) [82]. Given that PD presents with morbidity, which is more prevalent in the elderly, a novel treatment strategy to combat PD-associated symptoms is required.

CRISPR-Cas9 is increasingly being applied to treat neurodegenerative diseases, such as Parkinson’s disease (PD) and Huntington’s disease (HD) [86]. In a report by Klein and Westernberger, 28-well defined chromosomal locations which are convincingly related to PD, were presented, while six of those specific locations carry genome sequences with mutations that are confirmed to lead to monogenic PD, as a result of a single gene mutation [87]. Other studies, however, have pointed to a link between mitochondrial dysfunction and age-related pathogenesis of PD [88, 89]. In fact, a mutation in the protein PARKIN, at the cytosolic E3 ligase, that coordinates with PINK1 (PTEN-induced kinase 1) to facilitate the proteasomal clearance of dysfunctional mitochondria via the autophagy/lysosome pathway (mitophagy), was shown to cause accumulation of damaged and dysfunctional mitochondria in neurons in familial PD [90]. Potting et al, reported that the transcriptional repressor THAP11 negatively regulated endogenous PARKIN expression and demonstrated that knocking out the THAP 11 gene via CRISPR-Cas9 promotes phosphorylated ubiquitin (pUb) accumulation on mitochondria and induces the mitophagy activity for selective clearance of dysfunctional mitochondria [91].

Telomere shortening associated with mitochondrial dysfunction [92, 93] and DNA damage culminates in the activation of the tumor suppressor, p53 and in suppression of the key mitochondrial regulators, PGC-1α and PGC-1β, in aged individuals [92, 93]. Scheffold et al, showed that telomere shortening and mutation accelerate the aggregation of proteins, such as α-synuclein, in Lewy bodies and Lewy neurites that cause neuroinflammation in PD [94]. Given that CRISPR-Cas9 provides high-efficiency transgene insertion and deletion in mitochondrial DNA, the CRISPR-Cas9 system may be employed to study mitochondrial dysfunction for a better understanding of PD pathology, development of early diagnostic markers, and effective therapy targeting PD [70]. Co-injection of Cas9/gRNAs with exogenous donor target DNA fragments carrying original sequences can replace the mutant genes via NHEJ/HDR and can act as a powerful approach to treat PD patients [95]. Moreover, a novel two-step CRISPR-Cas9 genome editing strategy was discovered in telomerase biology to introduce precise modifications at endogenous mutant telomerase reverse transcriptase proteins (TERT) locus that regulate the telomerase activity in PD patients [96].

In terms of viral vectors, AAV vectors are considered the most successful tools for therapy of neurological disorders [97]. Given that PD patients are experiencing progressive loss of dopaminergic neurons in several brain regions including the substantia nigra and the putamen, clinical treatment for PD by the intraparen-chymal release of viral vectors has been classified into two categories which facilitate neuronal survival and function [97]. The first category induces the over-expression of enzymes that regulate neurotransmitter synthesis [97]. The second category overexpresses neurotrophic factors that facilitate neuron survival [97]. Kaplitt et al, introduced a gene therapy approach that transfers the glutamic acid decarboxylase (GAD) gene with AAV into neurons of the human subthalamic nucleus, catalyzing the production of γ-aminobutyric acid (GABA), a major inhibitory neurotransmitter in the brain, which reduces excessive excitatory glutamate output to target sites [98]. Following the same line of therapeutic approaches, AAV containing aromatic l-amino acid decarboxylase (AADC) gene, which converts levodopa into dopamine, was directly delivered into the putamen of PD patients, thereby reducing motor fluctuations [99]. In another therapeutic approach, the naturally occurring functional analog of the glial cell line-derived neurotrophic factor called Neuturin (NTN), was introduced by an adeno-virus based vector, enhancing dopaminergic neuron survival and nigrostriatal functions [100]. Recently, adeno virus-associated delivery of short hairpin RNA (shRNA) was used to target and knock down an endogenous *Snca* transcript as well as α-synuclein protein aggregation, leading to attenuation of the progressive motor deficits in PD patients [101]. Another potential therapeutic target is leucine-rich repeat kinase 2 (LRRK2), a significant cause for familial PD when presenting with a p.G2019S mutation [102]. Mutations within the α-synuclein encoding gene, SNCA, presents as early onset PD in patients [103].The CRISPR-Cas9 system has the potential to ameliorate or treat PD-associated pathology. With the capacity to study and clear dysfunctional mitochondria, insert functional genes to treat PD-associated symptoms, and knockdown transcripts responsible for observed PD-symptoms.

Commonly misconstrued as either a variant of AD or PD, or misdiagnosed as AD or PD, Lewy body Dementia (LBD) is one of the most common causes of dementia resulting from excessive and abnormal α-synuclein deposits in the brain called Lewy bodies and is associated with cognitive aging (LB) [104-106]. Hyper-phosphorylation of α-synuclein (SNCA) at the Ser-129 position is potentially the major modification responsible for the formation of LBs [107]. Deposition of LB in neurites, called LB neurites, leads to the observed disease symptoms which range from behavioral changes to cognitive disorders [104]. The disease onset is typically after age 50 and is more common in males than females; it is also estimated to be inherited in about 36% of the cases [104]. Though the symptoms are sometimes worse than AD, less attention has been devoted towards LBD, mainly because of misdiagnosis and the misunderstanding that LBD is a variant of Alzheimer’s or Parkinson’s disease [104]. Gene therapy approaches for LBD are based on the usage of viral vectors modulating the expression of neurotropic factors, anti-apoptotic genes, or dopaminergic enzymes; with candidate genes for therapy currently under investigation [104, 108-110]. So far, neither LBD nor the implementation of CRISPR-Cas9 technology as a therapeutic approach for LBD has been researched sufficiently. Further investigation of candidate genes and their physiological functions need to be initiated before the CRISPR-Cas9 system can be considered as an appropriate and effective therapeutic tool against LBD.

## Huntington's disease (HD) and gene therapy using the CRISPR-Cas9 system

Huntington’s disease (HD) is a dominantly inherited fatal genetic neurodegenerative disorder with an average age at onset of 40 years [111-113]. In HD patients, the basal ganglia and cerebral cortex are most typically affected parts of the brain [114]. The characteristic symptoms of HD are chronic and progressive involuntary choreiform movements, cognitive impairment, mood disorders, and behavioral changes [112]. HD is strongly related to biological age with the trinucleotide repeats expanding with age, but repeat size is not a good predictor of age of disease onset [112]. Moreover, it has been reported that the disease accelerates epigenetic aging with an increase in biological age by about 3.2 years [112]. Genetic alterations in the *huntingtin HD* (*HTT*) gene, which is located on chromosome 4p16.3, leads to an increase in the number of CAG trinucleotides repeats, causing the disease [112]. Repeats of 40 trinucleotidesor larger are associated with disease manifestation, repeats of 27-35 may not be associated with disease manifestation, but the number of repeats may expand further after parental transmission and cause disease in descendants [112]. However, repeats of less or equal to 26 are normal [112]. The resulting polyglutamine domain (polyQ) in the huntingtin protein (htt) encoded from the mutated *HD* gene induces conformational changes in the protein, leading to the formation of intracellular aggregates in the nucleus or sometimes outside the nucleus [115]. In terms of cellular mechanisms linked to aging, studies show that polyQ proteins impair the ubiquitin-proteasomal system, inducing the autophagy pathway [116-119]. It has been demonstrated that genetic modification of the *HD* gene can resolve the HD-phenotypes by eliminating its expression, which may provide a promising avenue for therapeutic strategies for HD [120, 121]. RNAi, Zinc finger nucleases, and antisense nucleotides have been reported to be beneficial for reducing the mutated *HTT* gene using different mouse models [120]. However, these approaches would require long term and repeated administration to provide a sustained reduction in the mutant gene [122, 123]. The CRISPR-Cas9 system of gene editing, on the other hand can be an exciting alternative methodology because of its potency and sequence specificity [122, 123]. Recently, an allele-specific genome editing system was developed for mutant *HTT*, targeting highly prevalent single nucleotide polymorphisms (SNPs) in the locus by CRISPR-Cas9, which more effectively reduces the mutant *HTT* expression *in vitro* as well as *in vivo* [120].

## The use of CRISPR-Cas gene therapy in colorectal cancer biology

As somatic and epigenetic mutations are tied to many of the hallmarks of cancer, the advancement and use of genome editing tools have been instrumental in the development of disease models and identification of oncogenes [124]. The commonly employed cancer treatment of chemotherapy is also used in the treatment of colorectal cancer [125]. Since the inception of homologous recombination techniques to modify genes, experimental advancements such as the use of site-specific endonucleases (ZFNs and TALENs ) have increased the precision of gene editing techniques; however, due to the cost and difficulty of producing these endonucleases, their usage has been limited [126-128]. Following its discovery as a tool for rapid, site-specific DNA cleavage, the prokaryotic immune system CRISPR /Cas9 has expanded the field of cancer biology significantly [33].

Since the completion of the Human Genome Project, the prospect of using genome-wide screens to understand the genetic components of normal physiology and disease have become more realistic. In yeast, the concept of “essential” and “non-essential” genes have been investigated to understand which genes, if knocked down, have a negative effect on cellular proliferation [129]. It has also been reported that the need for certain genes is context-dependent rather than universal [130]. In diseases such as cancer, this idea may be translatable into therapeutics - as genes essential to tumor biology, but non-essential to surrounding tissues make promising drug targets. Previously, the reliability of these screens was technologically limited, as RNAi for gene knockdowns often results in incomplete silencing and thus inconclusive results. To address this, a library of gRNA sequences targeting protein-coding genes was created, and using colorectal carcinoma (such as *KRAS*-activated (G13D) mutants DLD1 and HCT116) and other cancer cell lines expressing Cas9, over 5-fold more essential genes were observed when compared to RNAi or previous generation CRISPR screens [35]. After defining core fitness genes which are necessary across all cell lines, DLD1/HCT116-specific genes were identified to select potential therapeutic targets [35]. Interestingly, it was observed that the epidermal growth factor (EGF) signaling pathway was a strong hit in DLD1, but not in HCT116 cells [35]. DLD1 cells also showed selectivity to the EGF receptor inhibitor erlotinib [35]. An upstream *TP53* mutation in DLD1 cells was hypothesized to be involved in EGF dependence, as other cell lines carrying both the *KRAS* and the *TP53* mutations also showed erlotinib sensitivity; highlighting the idea that therapeutic options may be significantly different even between cell subtypes [35]. This was expanded on by characterizing metabolic phenotypes of *KRAS*-mutant colorectal carcinomas [36]. Mutations in *TP53* promote the malignant potential of colorectal cancer during the late phase of carcinogenesis, providing a potential therapeutic target for colorectal cancer [131]. By transducing HCT116 cells of wild-type or activated (G13D) *KRAS* with a sgRNA library targeting protein-coding genes and injecting them into mice, tumor xenografts were obtained and used to determine *KRAS*-lethal and enhancing mutations as well as for pathway analysis [36]. Of the genes enriched, which serve as potential targets for suppressive therapy, *INO80C* was selectively enriched in *KRAS*-mutant cells [36]. Probing datasets from The Cancer Genome Atlas showed an association between *INO80C* deletion and a worse prognosis for *KRAS*-mutant colorectal cancers. INO80 is a protein complex involved in maintaining genome stability, and deletion of the INO80C component has been shown to alter metabolic processes [132]. In the same vein, small-molecule inhibitors of metabolic pathways, particularly pathways where the NAD+ kinase and hepatic fructokinase (KHK) play a role, more strongly inhibit the growth of *KRAS*-mutant xenografts than wild-type [36]. Both kinases function to reduce reactive oxygen species (ROS) levels, and to create an energy-rich microenvironment favorable for proliferation [36].

Other survival pathways have also been studied in colorectal cancers. Recently, partitioning defective 3-like protein (Par3L) was identified as a stem cell maintenance protein and was observed to inhibit the kinase activity of the tumor suppressor protein Lkb1 [133]. To understand its potential role in cancer biology, Par3L-knockout CaCO-2 cells were created using a CRISPR-Cas9 system [38]. Par3L knockout resulted in lower proliferation and higher cell death when compared to wild-type cells [38]. Par3L-knockout cells were also more sensitive to chemotherapeutics, such as cisplatin, doxorubicin, and 5-fluorouracil [38]. Also, knocking down Lkb1 could partially recover cell death caused by Par3L deletion, suggesting that suppression of the Lkb1/AMP-activated protein kinase (AMPK) pathway is necessary for colorectal cancer cell survival, and that Par3L inhibition may be a novel therapeutic avenue [38].

In addition to their use as therapeutic target discovery tools, CRISPR-Cas9 systems can be used to study pharmacological tumor suppressor reactivation [37]. The E3 ubiquitin ligase Mdm2 is a key inhibitor of the tumor suppressor protein p53, repressing its transcriptional activity and target it for proteasome degradation [134, 135]. Re-activation of p53 is a promising broad-spectrum therapeutic avenue, as compounds such as nutlins and Reactivation of p53 and Induction of Tumor cell Apoptosis (RITA) have been the subject of several studies [136, 137]. While both inhibitors disrupt the p53-Mdm2 interaction, they bind differently. Nutlins occupy the p53 binding site in Mdm2, whereas RITA binds to the N-terminus of p53 [136-138]. It was hypothesized that the anti-tumor activities of these inhibitors might depend on the presence of wild-type p53 [37]. Using the CRISPR-Cas9-mediated gene editing system, a frameshift mutation was introduced into wild-type p53, which was then transfected to HCT116 cells [137]. The resistance to nutlin/RITA was compared to p53^-/-^ clones as well as with RNAi-downregulated p53 [137]. Surprisingly, it was shown that disruption of p53 resulted in the loss of nutlin response, whereas the RITA response was unaffected, suggesting that both drugs have significantly different mechanisms of action, while still targeting the same protein complex [137]. A commonly employed colorectal cancer mice model exploits the deletion of tumor suppressor gene, *Apc,* providing a target for the development of murine tumor organoids via CRISPR-Cas9, useful for assessing the function of genes in the context of tumorigenesis [139]. In summary, these studies showcased the power of CRISPR-Cas9 in dissecting the mechanisms of p53-reactivating compounds (Fig. 2).

## The use of CRISPR-Cas9 gene therapy in prostate cancer

Prostate cancer is the 6^th^ leading cause of cancer death and the 2^nd^ most commonly diagnosed cancer in men worldwide [140] (Fig. 1). At an early stage, prostate cancer may not cause any symptoms, however during the late stages the illness may show up as painful urination, decreased force in urination, erectile dysfunction and blood in semen amongst some symptoms [140, 141]. Commonly used cancer treatment options, such as chemotherapy, is also used to treat prostate cancer [142]. Member of the steroid hormone nuclear receptor family and a ligand-dependent transcription factor, the androgen receptor (AR) can be induced by binding of a ligand [143, 144]. Variants of the AR are constitutively expressed in prostate cancer cells and control the activity of cell proliferation, migration, apoptosis and invasion [145]. A recent report showed that the CRISPR-Cas9 technique of targeted androgen receptor disruption could successfully block the growth of prostate cancer cells [146]. GPRC6A is a G-protein coupled receptor that senses nutrition and is activated *in vitro* by several ligands like amino acids, calcium, zinc, osteocalcin (OC), and testosterone and has been implicated in prostate cancer [147]. Further in a human xenograft model, CRISPR-Cas9 targeting of GPRC6A suppresses prostate cancer tumorigenesis [148]. In another study, androgen receptor signaling was found to upregulate NANOG mRNA and protein, directly contributing towards prostate cancer [149]. Additionally, in a 2015 report, CRISPR-Cas9-mediated gene knockout of NANOG and NANOGP8 was observed to decrease the malignant potential of prostate cancer cells [150]. Researchers have also shown that Dickkopf-related protein 3 (DKK3) stimulates proliferation and differentiation in benign prostate cancer cells [151]. Cancer cells present PD-1, a checkpoint inhibitor, at their surface which inhibits T-cell mediated cancer clearance upon binding to an activated T-cell PD-1 receptor [152]. Therefore CRISPR-Cas9 mediated deletion of PD-1 may potentially enhance the effects of cytotoxicity and T-cell immune responses against cancer, with clinical trials underway to ascertain if this is a viable strategy against prostate cancer [152]. Lastly, a recent study showed that CRISPR-Cas9 mediated activation of DKK3 attenuates TGF beta signaling, thereby attenuating migration and proliferation in prostate cancer cells [153]. Overall, these studies showed the diverse use of CRISPR-Cas9 for several therapeutic strategies by utilizing genes known to contribute to prostate cancer (Fig. 2).

## The use of CRISPR-Cas9 gene therapy in breast cancer

Breast cancer is the second most common lethal adenocarcinoma among women [154]. According to the American Cancer Society, over 200,000 new invasive cases and 40,920 deaths are predicted for 2018 [155]. Breast cancer is divided into five molecular subtypes based on the presence of hormone (estrogen and progesterone), receptors (ER/PR), and human epidermal growth factor receptor 2 (HER) [156, 157]. These subtypes are defined as luminal A, luminal B, HER2 enriched, claudin-low, and triple-negative/ basal-like breast cancer (TNBC) [156, 157]. The main cause of breast cancer is considered to be mutations that over-activate cell signaling pathways and their effects on cell growth, proliferation and differentiation [158]. These mutations and their outcomes vary across individuals; for this reason, new treatments focusing on personalized medicine and CRISPR-Cas9 mediated genome editing are being investigated as potential therapeutic strategies [159]. In this section, the usage of CRISPR-Cas9 on somatic gene editing and transcriptional regulation is explored. In cancer genetics, somatic mutations are divided into two groups; driver mutations and passenger mutations [160]. Driver mutations induce neoplasia and are the main promoters of cancer progression [160]. On the other hand, passenger mutations have a neutral effect on cell growth [160]. Studies have shown the effect of driver mutations on specific genes by using the CRISPR-Cas9 gene-editing system [161]. In 2016, the effect of the tumor suppressor gene *Pten*, on invasive lobular breast cancer (ILC) was examined in mice [161]. *Pten* was silenced by CRISPR-Cas9 and lentiviral delivery of a *Pten* targeting single guide RNA (sgRNA) [161]. Usage of the Cas9-encoding lentiviruses resulted in Cas9-specific immune responses and tumor development differing from those observed in ILC [161]. However, in a lentiviral delivery of a *Pten* targeting single-guide RNA, invasive lobular breast cancer development was observed [161]. In another study, mitochondrial calcium regulator (MCU) was silenced by CRISPR-Cas9 [162]. This MCU downregulation reduced cell growth and prevented metastasis in TNBC [162]. CRISPR-Cas9 mediated targeting of oncogene *HER2* inhibits tumorigenicity and cell growth in breast cancer cells [163].

Cell-specific transcriptional regulation is one of the key factors in the development of cancer [164, 165]. For this reason, future personalized medicine may aim to target transcription factors. In the last five years, the activities of embryonic transcriptional factors have been detected during cancer progression [164, 166]. Most recently, the zinc finger protein Snail1 was examined in TNBC [166]. Snail1 was silenced using the CRISPR-Cas9 system in Hs578T cell lines, resulting in the dysregulation of several hundred genes and a reduction in the proliferation and invasive capacity of the cells [166]. In another study, cyclin-dependent kinase 7 (CDK7) was silenced in TNBC and its effect on the TNBC specific gene cluster Achilles cluster was examined [167]. The silencing of CDK7 induced apoptosis and impaired cell growth, in addition to downregulating several embryonic transcription factors such as EGFR or Sox2 [167]. Epigenetic modifications play a critical role in the regulation of transcription [168]. Recently, the ER-regulator gene, Src-1, was found to cause aberrant methylation of genes involved in cellular differentiation and development, in both patient tumors and endocrine-resistant models of breast cancer cells, directly suppressing the genes [168].

Overexpression of Src-1 resulted in hypermethylation induced downregulation of five target genes NTRK2, NR2F2, CTDP1, SETBP1 and POU3F2 [168]. In cell lines, the downregulation of these genes conferred an increased ability for self-renewal and invasive-metastatic features [168]. When Src-1 knock-out by CRISPR-Cas9 or demethylation treatment was performed, the target genes were instead up-regulated, and the cell lines showed a reduced capacity for self-renewal, colony formation, and a renewed sensitivity to the endocrine treatment [168]. When treating ER positive breast cancers, ER is frequently targeted in hormone therapy, although cancers may retain expression of ER while simultaneously developing resistance to the hormone therapy [169]. Therefore, the discovery of mediators of ER function susceptible to therapeutic intervention may increase the efficacy of any therapeutics targeting the ER [169]. Cyclin-dependent kinase 8 (CDK8), an oncogenic transcriptional regulator, and CDK19 are negative prognostic markers in breast cancer [169]. Knockout of CDK8/19 via CRISPR-Cas9 inhibits ER positive breast cancer cell estrogen-induced transcription [169]. An overexpression of UBR5 is recorded in TNBC cells, is associated with poor patient survival, and is dysregulated in multiple types of cancer [170]. This suggests that UBR5 may play a critical role in breast cancer aggressiveness [170]. CRISPR-Cas9 mediated knockout of UBR5 in the murine 4T1 breast cancer cell line increased apoptosis, suppressing tumor growth [170]. The oncogene Migration and Invasion Enhancer (MIEN1) facilitates cancer cell mobility and progression [171]. Expression of MIEN1 in breast cancer cells was arrested after the CRISPR-Cas9 mediated introduction of certain genomic deletions in MIEN1 [171]. In another study, the catalytic domain (CD) of a Ten-Eleven Translocation (TET) methylcytosine dioxygenase1 (TET1CD) was fused with deactivated Cas9 [172]. The fusion protein was able to demethylate specific regions of the *BRCA1* gene in MCF-7 and HeLa cell lines, and it reduces cell viability, cell growth, and chemoresistance [172]. Breast cancer can be initiated from mutations of many steps during the cell cycle and progress to metastatic disease. Overall, these studies showed that using CRISPR-Cas9 interference method can be a potential therapeutic approach in multiple molecular subtypes of the disease (Fig. 2).

## Therapeutic potential of CRISPR-Cas9 in the treatment of ovarian cancer

Among women, ovarian cancer (OC) is the 7^th^ most frequently diagnosed cancer and may be inherited as a result of moderate to highly penetrant rare mutations, increasing genetic susceptibility to OC [173]. The risk of a woman developing OC within her lifetime is 1 in 75, with a 1 in 100 chance of mortality [173]. Worldwide, OC is annually responsible for 152,000 deaths and 239,000 new cases [173]. Mutations and overexpression of oncogenes have been linked to OC tumor development [173, 174]. Therefore, the use of the CRISPR-Cas9 system to target and silence the OC-associated oncogenes may potentially ameliorate the observed pathology in this targeted therapeutic strategy.

One such oncogene, DNA methyltransferase 1 (DNMT1), inactivates tumor suppressor genes when overexpressed and is crucial in the maintenance of cancer stem cells, and correlates with OC relapse and tumorigenesis [175, 176]. CRISPR-Cas9 targeting of DNMT1 was found to downregulate expression of DNMT1 and inhibit the rate of tumor growth by 84%, demonstrating the potential of DNMT1 as an OC therapeutic target [175, 176]. Overexpression of MutT Homolog1 (MTH1) has been detected in various cancer populations [177]. Apoptosis and increased genomic damage of the cancerous cells has been observed via knockout of MTH1 expression, whereas healthy cells were unaffected by this treatment [177]. CRISPR-Cas9 targeting of the MTH1 gene resulted in inhibition of MTH1 expression and significantly inhibited tumor growth, responsible for inducing approximately 64% of the total apoptotic ratio of human ovarian cancer cells [177].

The CRISPR-Cas9 system is a powerful tool used in loss of function studies [178]. One such study demonstrated that the addition of mutations in the pre-miRNA hairpin sequence of disruption of miR-21, an miRNA found to contribute to chemoresistance and tumorigenesis and is upregulated in cancers, resulted in the inhibition of the epithelial-to-mesenchymal transition (EMT) in ovarian cancer cells [178]. In another study, poly (ADP-ribose) polymerase-1 (PARP-1), involved in responding to DNA damage, regulation of the cell cycle, and apoptosis, was investigated as a potential therapeutic target using the CRISPR-Cas9 system [179]. Inhibition of PARP-1 results in cancer cell death [179]. CRISP-Cas9-mediated inhibition of PARP-1 in ovarian cancer cell line SKOV3 induced apoptosis, restricting the proliferation of the cancer cells [179]. In summary, these studies highlight the potential of the CRISPR-Cas9 system as a therapeutic tool to treat OC.

## CRISPR-Cas9 and Inflammatory disorders

### (1) Rheumatoid arthritis

Rheumatoid arthritis (RA) is an inflammatory disease and autoimmune disorder that rarely coexists with Progressive multifocal leukoencephalopathy (PML) resulting in disability and impaired movement [180, 181]. PML is a central nervous system disorder associated with demyelination and viral replication in the brain as a result of JC virus (JCV) infection and viral transcription of the JCV promoter in a cell type-specific manner, in immuno-compromised patients with neurologic complications [182]. Rheumatoid arthritis may also occur with PML even in patients without malignancies or in patients with a positive Human Immunodeficiency Virus (HIV) status, albeit at very low incidence [180]. Although RA and PML can affect the non-elderly, the severity of the disease is most often seen in the elderly [183, 184]. Khalili et al have developed a CRISPR-Cas9 gene-editing strategy where transient or conditional expression of Cas9 and gRNAs specifically targets DNA sequences corresponding to the N-terminal region of the T-antigen [185]. By introducing the mutation, the gRNA alters the expression and function of viral protein, and thereby suppresses viral replication [185]. Another study involved MicroRNA 155 (miR-155), which is a key proinflammatory regulator in cases of RA [186]. CRISPR-Cas9-mediated miR-155 genome knockout in the RAW264.7 macrophage cell line, severely impaired production of proinflammatory cytokines, demonstrating its potential as a therapeutic strategy against RA [186]. Autoimmune disease treatment may be complemented with adaptive regulatory T-cell (Treg) therapy [181]. Enhancement of the *in vivo* plasticity and stability of Treg cells is critical for the improvement of this therapeutic strategy [181]. The transcription factor forkhead box P3 (FOXP3) prevents pro-inflammatory gene expression and increases the expression of anti-inflammatory genes [181]. Tregs use 2 different mechanisms to suppress an autoimmune response: contact-dependent or contact-independent inhibition, resulting in either a reduction of T-cell proliferation or secretion of cytolytic proteins and anti-inflammatory cytokines, respectively [181]. Modification of FOXP3-associated genes, such as Stub1, PD-1, CTLA-4, and BACH2 among others, via CRISPR-Cas9 increases Treg stability [181]. This results in an improved Treg suppressive ability [181] (Fig. 2).

### (2) Lung infections

The respiratory tract is responsible for gas exchange. Inhalation of a sufficient concentration of microbial agents, allergens, or dust aerosols may lead to accumulation and a major pulmonary disease [187]. Human primary airway epithelial cells (AECs) are the first line of defense against hazardous inhaled environmental factors such as pathogens and pollutants [188]. Inhalation of the above particles activates the immune system to remove foreign particles from the respiratory tract [188]. Failure to remove foreign particles may result in excessive accumulation and induction of the inflammatory responses, resulting in swelling and blockage of the respiratory tract [188]. Therefore, it has become increasingly necessary to target the lung epithelial cells to introduce modifications making cells more resistant towards infections. Studies have demonstrated effective gene knockouts and sequence level nucleotide alterations in both human transformed cells and induced pluripotent stem cells (iPSCs) by the CRISPR-Cas9 gene-editing system [189, 190]. Even though currently, this technology has only been applied to a few primary human cell types, the application of CRISPR-Cas9 technology in reduction of primary airway epithelial cell infection is an active field in research.

In a recent study, lentiviral delivery of CRISPR-Cas9 machinery and conditional reprogramming culture methods was used to knockout the MUC18 gene, which plays a pro-inflammatory role, in human primary nasal airway epithelial cells (AECs) [12]. MUC18 knockout cell populations showed that IL-8 responses of AECs were significantly reduced in the absence of functional MUC18 protein [12]. This led to a reduction of inflammation during bacterial infection [12]. Moreover, CRISPR-Cas9 mediated gene knockout was applied to study other genes in primary human airway epithelial cells and potentially other primary cell types. Wu et al. used the CRISPR-Cas9 system to correct a dominant cataract-causing mutation in the *Crygc* gene, demonstrating that the gene could be rescued by targeting the mutant allele with a sgRNA co-injected with Cas9 mRNA into zygotes [43]. In a study by De Ravin et al. they used CRISPR to correct blood stem cells from patients with a NOX2-induced immunodeficiency disorder (chronic granulomatous disease) and engraft the CRISPR-repaired human stem cells in mice which differentiate into leukocytes expressing a functioning NOX2 protein [42]. Overall, these studies show that disruption of target genes via the usage of the CRISPR-Cas9 gene editing system can be a promising therapeutic tool against inflammatory diseases (Fig. 2).

## Knowledge Gap

Although there have been a few studies investigating the potential efficacy of the CRISPR-Cas9 system as a therapeutic tool to combat age-associated disorders, more research is needed before this gene-editing system can be implemented in humans. First, further candidate gene studies are required, as some of these disorders are not monogenic, possibly requiring the editing of multiple genes, for successful treatment. It is also critical for the gRNAs to target select genes with minimal sequence overlap with other genomic loci [191]. Secondly, a method to safely and efficiently deliver the CRISPR-Cas9 system is needed, especially when targeting neuronal cells past the blood brain barrier. As implementation of AAVs may result in an immunogenic response, cytotoxicity, long-term expression, and off-target effects [192]. Concerns also extend to the risk of retroviral or lentiviral vectors possibly integrating near tumor suppressors or oncogenes, potentially resulting in the deactivation of those genes and promoting oncogenesis [191]. Additionally, the appropriate dosage of CRISPR-Cas9 must be investigated, as studies have demonstrated that a decreased dosage of CRISPR-Cas9 results in reduced off-target gene editing [191]. This implies that the high degree of reported off-target effects may be as a result of high CRISPR-Cas9 dosage. The dosage of Cas9 has been demonstrated to alter both its specificity and kinetics, with a 2-fold drop in on-target efficiency alongside a 7-fold increase in the specificity ratio when the Cas9 dosage is decreased by 5-fold during plasmid transfection [193]. Additional study investigated the possibility of a dose-dependent effect of the gRNA with the Cas9 protein on channel catfish (*Ictalurus punctatus)* mutation rate and embryonic survival [194]. The mutation frequency of the embryos increased with greater dosages of gRNA/Cas9 [194]. Higher dosages of gRNA/Cas9 also resulted in decreased hatching percentage and increased mortality [194]. More research is needed to elucidate the relationship between CRISPR-Cas9 dosage and off-target effects. Lastly, Investigators must also consider if the treatment will lead to a significant reduction in the patient’s observed pathology. For example, PSEN1, PSEN2, APP, and APOE4 are potential therapeutic targets for treating AD patients, using the CRISPR-Cas9 system. However, these genes solely have a strong-association with AD, not necessarily a cause for the disease. Thus, inhibition of these genes will not necessarily prevent the observed AD-associated symptoms. This suggests that further research into candidate genes and murine CRISPR-Cas9-mediated gene-knockout studies are needed before this gene editing tool is implemented in human studies. Overall, further research resolving the abovementioned issues is required before employing the CRISPR-Cas9 gene editing system as a therapeutic tool in human clinical studies.

## Conclusion

The use of CRISPR-Cas9 for genome-wide studies have enabled and expanded the nature and utility of genetic screens in humans to correct and precisely modify the genome and represents a potential means of correcting disease-causing mutations [195]. Although still in its infancy, the CRISPR-Cas9 system has revolutionized the studies of gene-function and is making a huge impact on genetic therapy in human health. In comparison with previous gene modulation techniques such as RNAi, the use of CRISPR-Cas9 is more efficient and highly specific. Furthermore, the CRISPR-Cas9 System has become a potent gene-editing tool capable of correcting gene-mediated age-related pathology. Deleting the hexanucleotide repeat expansions in the *C9ORF72* gene using the CRISPR-Cas9 system or correcting the *SOD1* or *FUS* gene mutations may ameliorate non-familiar ALS and FTD, or FALS respectively (Fig. 2). Early-onset AD may be treated via correcting mutations in *PSEN1*, *PSEN2*, and *APP*, reducing beta-amyloid generation. Whereas a mitochondria-targeted CRISPR-Cas9 could be employed to revert or remove mutations which accumulate with age. Mitochondrial dysfunction-induced PD may be treated by replacing the mutant genes with the original sequences thus preventing α-synuclein protein accumulation in Lewy bodies and Lewy neurites, overexpression of neurotrophic factors facilitating neuron survival, or reducing patient motor fluctuations by delivering the AADC gene into the putamen of PD patients. Also, cancer cells may be targeted by the CRISPR-Cas9 system, with knockouts of Par3L, Src-1, and GPRC6A ameliorating colorectal, breast, and prostate cancer respectively, resulting in increased sensitivity to chemotherapeutics, lower proliferation, and higher cancer cell death. During infection, secretion of interleukin-1 serves as a pro-inflammatory cytokine in tissues, preventing stem cell differentiation and promoting aggressive tissue degradation, resulting in tissue damage [196]. Upon high levels of immune system secretion of inflammatory molecules, it becomes imperative to target these molecules. In addition to targeting IL-1, another strategy involves designing inflammation-resistant induced pluripotent stem cells (iPSCs) by knocking-out the IL-1 signaling pathway. In this scenario CRISPR-Cas9 plays a promising role as this system has been used widely to create engineered eukaryotic cells with either loss-of-function or gain-of-function alterations [9, 41]. Similar studies have been done on zebrafish cells, tumor cell lines and primary dendritic cells [34, 197]. Therefore, the role of CRISPR-Cas9 modulation seems promising in targeting inflammation, especially in diseased and damaged tissues. Reducing pro-inflammatory cytokine production through miR-155 knockout holds promise as a therapeutic strategy for both RA and inflammation. Whereas, knockout of MUC18, in AECs, significantly reduced inflammation and may result in reduced swelling and blockage of the respiratory tract. However, this therapeutic technology is far from being clinically approved by the FDA due to related challenges and limitations (Summarized in Table 1), such as the off-target effects, transfection efficiency, and short half-life [9-13, 15]. If clinical use is achieved, the CRISPR-Cas9 gene-editing system will ameliorate aging-associated pathology, affecting numerous diseases, reducing disease burden, morbidity, and mortality.
